# Transcriptome profiling of *Elymus sibiricus,* an important forage grass in Qinghai-Tibet plateau, reveals novel insights into candidate genes that potentially connected to seed shattering

**DOI:** 10.1186/s12870-017-1026-2

**Published:** 2017-04-21

**Authors:** Wengang Xie, Junchao Zhang, Xuhong Zhao, Zongyu Zhang, Yanrong Wang

**Affiliations:** State Key Laboratory of Grassland Agro-ecosystems, College of Pastoral Agriculture Science and Technology, Lanzhou University, Lanzhou, China

**Keywords:** *Elymus sibiricus*, Seed shattering, Abscission layers, Next-generation sequencing, Transcriptome analysis, Mechanism

## Abstract

**Background:**

*Elymus sibiricus* is an important forage grass in semi-arid regions, but it is difficult to grow for commercial seed production due to high seed shattering. To better understand the underlying mechanism and explore the putative genes related to seed shattering, we conducted a combination of morphological, histological, physiochemical and transcriptome analysis on two *E. sibiricus* genotypes (XH09 and ZhN03) that have contrasting seed shattering.

**Results:**

The results show that seed shattering is generally caused by a degradation of the abscission layer. Early degradation of abscission layers was associated with the increased seed shattering in high seed shattering genotype XH09. Two cell wall degrading enzymes, cellulase (CE) and polygalacturonase (PG), had different activity in the abscission zone, indicating their roles in differentiation of abscission layer. cDNA libraries from abscission zone tissue of XH09 and ZhN03 at 7 days, 21 days and 28 days after heading were constructed and sequenced. A total of 86,634 unigenes were annotated and 7110 differentially expressed transcripts (DETs) were predicted from “XH09-7 vs ZhN03-7”, “XH09-21 vs ZhN03-21” and “XH09-28 vs ZhN03-28”, corresponding to 2058 up-regulated and 5052 down-regulated unigenes. The expression profiles of 10 candidate transcripts involved in cell wall-degrading enzymes, lignin biosynthesis and phytohormone activity were validated using quantitative real-time PCR (qRT-PCR), 8 of which were up-regulated in low seed shattering genotype ZhN03, suggesting these genes may be associated with reduction of seed shattering.

**Conclusions:**

The expression data generated in this study provides an important resource for future molecular biological research in *E. sibiricus*.

**Electronic supplementary material:**

The online version of this article (doi:10.1186/s12870-017-1026-2) contains supplementary material, which is available to authorized users.

## Background

Seed shattering is thought to be an important adaptive trait for seed dispersal in wild plants, but is also a major cause of seed yield loss in many cereal crops [1]. Therefore, the loss of seed shattering is considered one of the key events in the process of most cereals’ domestication [2]. Along with other agronomic traits such as thousand grain weight, stress tolerance, and plant height, low seed shattering has been selected as an important agronomic trait in cereal breeding programs.

In cereal grasses, seed abscission occurs in the abscission zone (AZ), and the abscission pathway includes four major steps: abscission zone formation and development, response to abscission signals, activation of abscission, and differentiation of the abscission layer [3]. Previous studies showed seed shattering is generally caused by abscission, and seed retention results from loss of the abscission layers [4, 5]. The shattering habit is a complex polygenic trait that is controlled by many genes [2, 6]. In *Arabidopsis*, a MADS-box transcription factor gene *STK* and a bHLH transcription factor gene *HEC3* regulate the formation of seed AZs [7, 8]. In rice, several major quantitative trait locus (QTLs) and genes for seed shattering have been identified and cloned, including *SH4* [9]*, qSH1* [2], *OsCPL1* [10] and *SHAT1* [11]. *SH4* is a major seed shattering QTL and encodes a transcription factor with a Myb3 DNA binding domain and a nuclear localizing signal [9]. *qSH1* encodes a BEL1-type homeobox gene and regulates pedicel AZ formation, and an single nucleotide polymorphism (SNP) in the 5′ regulatory region of the *qSH1* gene causes loss of seed shattering owing to the absence of abscission layer formation [2]. Rice pedicel AZ formation is also regulated by *SHAT1* gene, which is a member of APETALA2 (AP2) family transcription factors [11]. The *OsCPL1* gene encodes a protein containing a conserved carboxy terminal domain (CTD) phosphatase domain, which represses differentiation of the abscission layer during panicle development [10]. Additionally, previous research revealed that a variety of genes involved in cell wall degradation and abscission-promoting phytohormone signaling are up-regulated during abscission [12, 13].

In comparison, studies of seed shattering in forage grasses are limited. In hybrid *Leymus* (Triticeae) wildryes, a major-effect seed retention QTL was identified [14]. A MSDS-box gene *WM8* was cloned in *Elymus nutans* [15]. However, the mechanism of seed shattering in many forage grasses remains largely unexplored and poorly understood. Breeding objectives of forage grasses mainly focus on forage quality, biomass yield, and stress tolerance while seed shattering is relatively unimportant to the end users. The seed shattering habit of many forage grasses has therefore received little attention from forage breeders, despite the fact that seed shattering is a commonly observed trait in many forage cultivars and wild grass species. Previous research has shown that increased seed retention did not influence forage quality, and suggested seed retention would be one of desirable traits in grass seed crops [4]. Selection for seed retention and improvement of seed shattering is critical for forage grasses with a high degree of seed shattering.

*Elymus sibiricus* (Siberian wild rye), the type species of the genus *Elymus*, is an economically important perennial cold-season, self-pollinating and allotetraploid forage grass, indigenous to northern Asia [16]. In Qinghai-Tibet Plateau, it is widely used in natural grasslands and cultivated pastures due to its stress tolerance, good forage quality, and adaptability to local environments with low temperature and high altitude [17]. Because of seed shattering, however, *E. sibiricus* is difficult to grow for commercial seed production. Within the provinces of Qinghai and Sichuan, China, where the vast majority of *E. sibiricus* seed (2,400,000 kg) is produced each year, the average seed yield is only 690 kg.ha^−1^ due to seed shattering. Indeed, seed shattering can cause up to 80% yield losses if harvesting is delayed due to adverse conditions [18]. In a previous study, we found wide variation in the tendency for seed shattering among a large spaced-planted population of *E. sibiricus*, and no significant correlation between seed shattering and other agronomic traits [19]. Those data suggested genetic variation for seed shattering and provided a suitable population from which the molecular mechanisms of seed shattering may be investigated. Although transcriptome analysis based on next-generation sequencing (NGS) has allowed for the elucidation of complex genetic regulatory networks and provided functional data for many genes related to important agronomic traits [20, 21], these tools and sequence resources for seed shattering in *E. sibiricus* are still lacking. This is the first step to investigate the mechanism of seed shattering for this species.

To dissect the mechanism that leads to seed shattering and explore the putative genes related to seed shattering in *E. sibiricus*, we conducted morphological, histological, and physiochemical measurements coupled with transcriptome analysis on a high seed shattering genotype (XH09) and low seed shattering genotype (ZhN03). The results of this study will lead to a better understanding of the mechanism of seed shattering and would be helpful for breeding improvement programs in seed retention for this species.

## Methods

### Plant materials and growth conditions

**Fig. 1:**
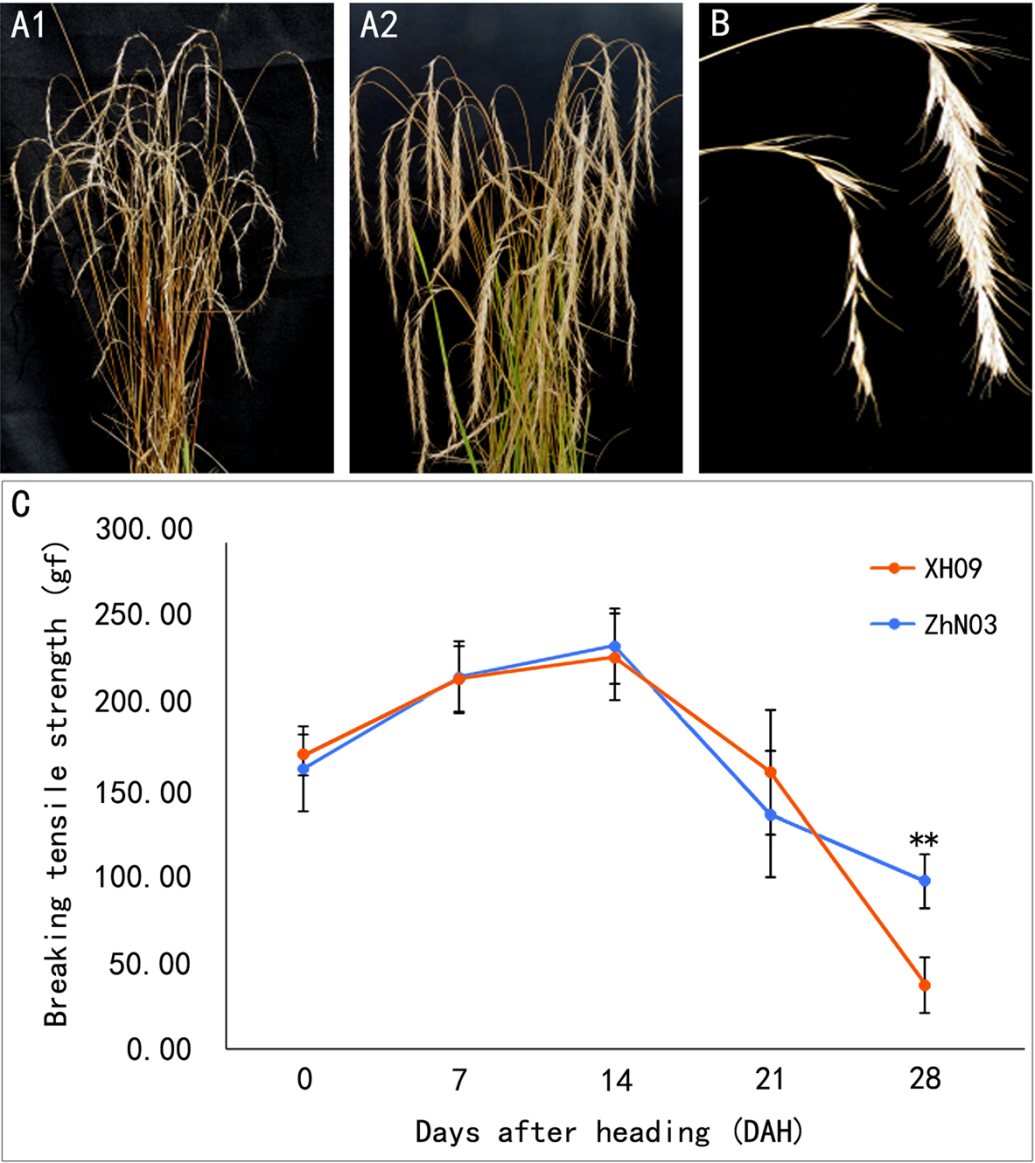
Different seed shattering habits of two *E. sibiricus* genotypes. (**a1**) High seed shattering type XH09. (**a2**) Low seed shattering type ZhN03. (**b**) Seed shattering degree of inflorescence in XH09 and ZhN03. Photos taken at 28 days after heading. (**c**) Time-course changes in the seed shattering degree of XH09 and ZhN03 at 7, 14, 21 and 28 days after heading. BTS was measured upon detachment of seed from the pedicels by pulling. Bars indicate the mean values ± standard deviation. Double asterisks (**) represent significant difference of BTS between XH09 and ZhN03 at *p* < 0.01 level

The plant materials consisted of two wild *E. sibiricus* genotypes XH09 and ZhN03 collected from Xiahe and Zhuoni, southern Gansu province, respectively (Fig. 1 a1, a2). *E. sibiricus* is not an endangered or protected species, thus, no permissions were required for collecting these samples in China. Formal identification of these samples is conducted in the State Key Laboratory of Grassland Agro-ecosystems, Gansu, China. Samples were identified based on some important phenotypical characteristics such as plant height, inflorescence, leaf, stem and seed. This species is small-anthered and long-awned bunchgrass. They were selected and used in present study based on a previous screening for seed shattering in 28 *E. sibiricus* accessions [19]. The seeds of two genotypes were germinated in plastic boxes with moistened blotter paper at room temperature. After germination, seedlings were grown under greenhouse conditions until they were 8 weeks old. Then they were transplanted to the field plots in the experimental station at Lanzhou University, Yuzhong, Gansu, China (latitude 35°34′ N, longitude 103° 34′ E, elevation 1720 m). No any permissions were required to carry out field experiment.

### Seed shattering phenotyping and histological analysis of pedicel structure

The inflorescence of *E. sibiricus* is a spike containing 15–30 spikelets. Each spikelet consists of 5–8 normally developed florets with long awn (Fig. 1b). The level of seed shattering of XH09 and ZhN03 was determined by measuring the breaking tensile strength (BTS) required to detach the seeds from the pedicels [9]. Thirty randomly chosen spikelets of each plant were examined at each of the five developmental stages, 0, 7, 14, 21, 28 days after heading (DAH), and their average BTS values were calculated. Histological analysis of pedicel structure was carried out at the same five development stages concurrent with seed shattering measurements.

In order to reduce variation due to the spikelet position at each developmental stage, the three central spikelets of each florescence were used, and within each spikelet, the central florets were dissected together with a part of the rachilla [4]. The pedicels of each accession were fixed in solution 60: 5: 5: 30 ethanol: acetic acid: formalin: water solution and stored at 4 °C in 15 M ethanol [4]. They were then dehydrated in a gradient of ethanol solutions (50, 70, 90 and 100%) for 60 min, respectively. After treatment with dimethylbenzene and a soaking in paraffin, tissue samples were sectioned longitudinally to a thickness of 8 μm, and stained for 3 min with Safranine-fast Green (Zhongtai, Shanghai, China). After staining, the pedicel structures were then observed under a Nikon Microphot FXA microscope (Nikon Corporation, Tokyo, Japan). Scanning electron microscopy was used to examine the pedicel junctions after detachment of seeds to detect the relationship between abscission layer development and seed shattering degree at each of the five developmental stages [2].

### Physiochemical analysis of the abscission zone

The abscission zone tissues of the two genotypes (XH09, ZhN03) were harvested according to methods described by Li et al. [9]. The enzyme activity of two cell wall-degrading enzymes (cellulase and polygalactouranase) was assayed in abscission zones of the two genotypes at the same five developmental stages used for BTS and histological analyses, following the manuscript’s protocol of plant CE ELISA kit and plant PG ELISA kit, respectively.

### RNA extraction, cDNA library construction, and RNA-seq

Abscission zone tissues of the two genotypes were collected at three of the five developmental stages: 7 days, 21 days and 28 days after heading (DAH). The three stages were selected based on results of seed shattering, histological and physiochemical analysis. According to our previous study, seed shattering was visible at 14 DAH, transcriptome changes should start before this time point, therefore, 7 DAH was used as “zero time” before seed shattering related genes are activated. Each collected flower-pedicel structure consisted of an approximately 1- mm region of the pedicel and 1.5 mm of the flower, which included the abscission zone [9, 22]. Approximately 30 mg of this abscission zone tissue was collected for each replicate. The test was carried out with three biological replicates. This material was immediately placed in liquid nitrogen and stored at −80 °C for later RNA extraction. Total RNA from each tissue was extracted using Plant total RNA Kit (TIANGEN, Beijing, China) according to the manufacturer’s instructions. RNA concentration and quality was measured using an Agilent 2100 Bioanalyzer (Agilent Technologies, Inc., Waldbronn, Germany). Total RNA samples were sent to Biomarker Technologies Corporation (Beijing, China) for cDNA library construction and transcriptome sequencing. Poly (A) mRNAs were enriched from the total RNA using magnetic oligo (dT) beads. RNA fragmentation, double-stranded cDNA synthesis, and PCR amplification were carried out according to the Illumina RNA-Seq protocol. Finally, sequencing of purified cDNA library were carried out on an Illumina GA-П (Illumina Inc., USA) using the Chrysalis 36 cycles v 3.0 sequencing kit, with one lane of 2 × 101 bp reads from both ends of the fragments (“paired ends”) with 180 bp insert distance for assembly.

### De novo assembly, and annotation

The clean reads were obtained after filtering adaptor sequences and reads with ambiguous ‘N’ bases and with a base quality less than Q30 using the FASTX toolkit. De novo transcriptome assembly of the quality reads was performed using the Trinity program [23]. Based on the Trinity assembly results, the unigene sequences were queried using BLASTX against the NCBI non-redundant protein sequence (Nr), Annotated protein sequence database (Swiss-Prot), Gene Ontology (GO), Protein family (Pfam), euKaryotic Orthologous Groups (KOG), Kyoto Encyclopedia of Genes and Genomes (KEGG), and Cluster of Orthologous Groups (COG) databases (E-value ≤1e-5) to retrieve homology-based protein functional annotations. GO terms regarding the biological process, molecular function and cellular component were assigned to each sequence annotated using the Blast2GO software [24]. The WEGO software was used to plot the distribution of GO annotations of transcripts [25].

### Analysis of the functional enrichment of differentially expressed transcripts (DETs)

Transcripts were mapped to the assembly using SOAPaligner, then the Fragments Per Kilobase per Million fragments mapped (FPKM) value for each transcript was measured according to methods described by Mortazavi et al. [26]. The transcript fold-change was calculated using the formula log_2_ (FC), and the correction for multiple tests used the false discovery rate (FDR) control method [27]. An absolute value of the log_2_ (FC) ≥ 2 and FDR significance score ≤ 0.01 were set as the thresholds to call significant DETs between two samples. STEM software was used to cluster the DETs with a *p* ≤ 0.05 [28], and GO enrichment analysis and KEGG pathway enrichment analysis of the DETs were performed using agriGO [29] and KOBAS 2.0 [30], respectively.

### Validation of RNA-seq data by quantitative real-time PCR (qRT-PCR)

A portion of total RNA used for the RNA-Seq analysis was used to make cDNA for qRT-PCR. qRT-PCR was conducted using the SYBR Premix Ex Taq™ II quantitative PCR system (Takara, Dalian), following the manufacturer’s instructions, and reactions occurred on a Bio-Rad iQ5 real-time PCR instrument (Bio-Rad, Hercules, CA, USA). Based on the transcriptome results, ten candidate genes involved in seed shattering were selected for the qRT-PCR assays. Gene-specific primers were designed using Primer Express software (Applied Biosystems) and are shown in Additional file 1: Table S1. Expression levels of these DETs were calculated relative to reference gene *GAPDH* using the 2^-ΔΔCt^ method [31]. All of the samples were tested in triplicate, and the experiments were performed on three biological replicates.

## Results

### Time-course change in seed shattering degree of two genotypes

The changes in the seed shattering degree of XH09 and ZhN03 were characterized over time by measuring pedicel breaking tensile strength (BTS), which is inversely proportional to shattering degree. During the first 14 days after heading (DAH), the BTS value did not differ between XH09 and ZhN03 and were maintained at more than 150 gf (Fig. 1c). Significantly different BTS values were found between XH09 and ZhN03 at 28 DAH. The BTS of ZhN03 began to decrease after 14 DAH, but remained above 90 gf at 28 DAH. In comparison, the BTS value of XH09 decreased quickly after 14 DAH, and dropped below 50 gf at 28 DAH. The seeds of XH09 were easily threshed by hand crushing. Therefore, wild accessions ZhN03 and XH09 can be characterized as low - and high - seed shattering, respectively.

### Histological and physiochemical analysis of abscission zone

**Fig. 2:**
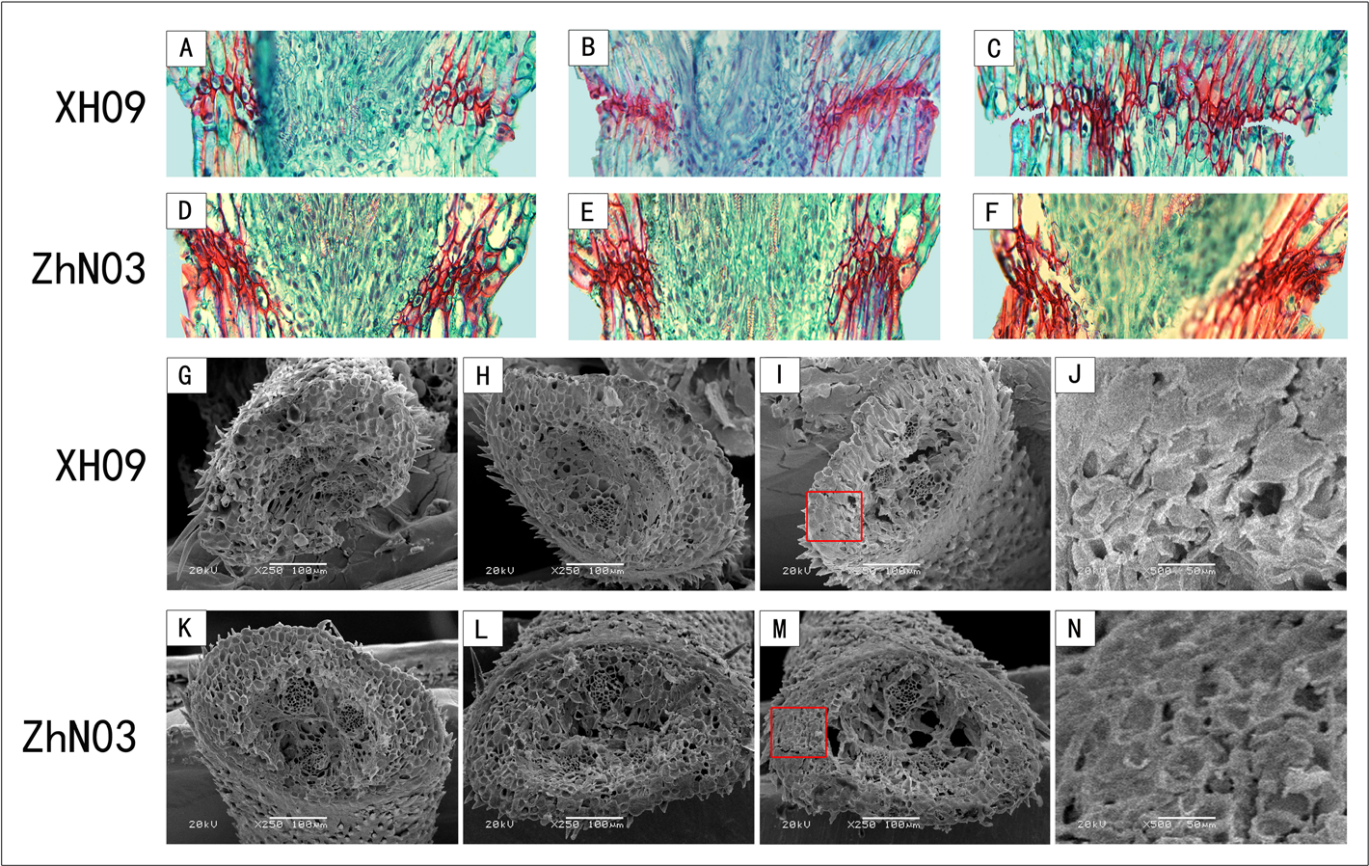
Histological analysis of abscission zone. (**a**) and (**d**), (**b**) and (**e**), (**c**) and (**f**) show longitudinal sections across the abscission zone of XH09 and ZhN03 at 7 DAH, 21 DAH and 28 DAH, respectively. Sections were stained with safranine-fast green, and lignin in red. (**g**) and (**k**), (**h**) and (**l**), (**i**) and (**m**) show scanning electron microscopy photos of pedicel junction after detachment of seeds in XH09 and ZhN03 at 7 DAH, 21 DAH and 28DAH, respectively. (**j**) and (**n**) show close-up scanning electron microscopy photos corresponding to red boxes in (**i**) and (**m**). A peeled-off and smooth surface is observed in the high seed shattering genotype XH09 (**j**), whereas broken and rough surface is observed in the low seed shattering genotype ZhN03 (**n**)

Anatomical investigation with longitudinal sections indicated abscission layers were already present at heading in XH09 and ZhN03. They occurred on both sides of the vascular bundle, which could be stained dark red by safranine. The cells of the abscission layer were smaller than the parenchyma cells in the rachilla, and had an elliptic shape and an organized position. Degradation of the abscission layer was not observed in two genotypes by 14 DAH. Degradation of the abscission layer occurred in XH09 at 21 DAH (Fig. 2b), and broken abscission layer was found at 28 DAH (Fig. 2c). In comparison, serious degradation of the abscission layer was not observed in ZhN03 at 21 (Fig. 2e) and 28 DAH (Fig. 2f). Early degradation of abscission layers was associated with the increased seed shattering in high seed shattering genotype XH09. Based on these staining results, there was less lignin in the abscission zone and surrounding pedicel tissues of XH09 (Fig. 2b) than in the ZhN03 (Fig. 2e). Additionally, scanning electron microscopy showed there was a smooth fracture surface on the rachilla in XH09 at 28 DAH (Fig. 2i, j) while in ZhN03 rough and irregular surface was observed, and cell structure was visible (Fig. 2m, n).

**Fig. 3:**
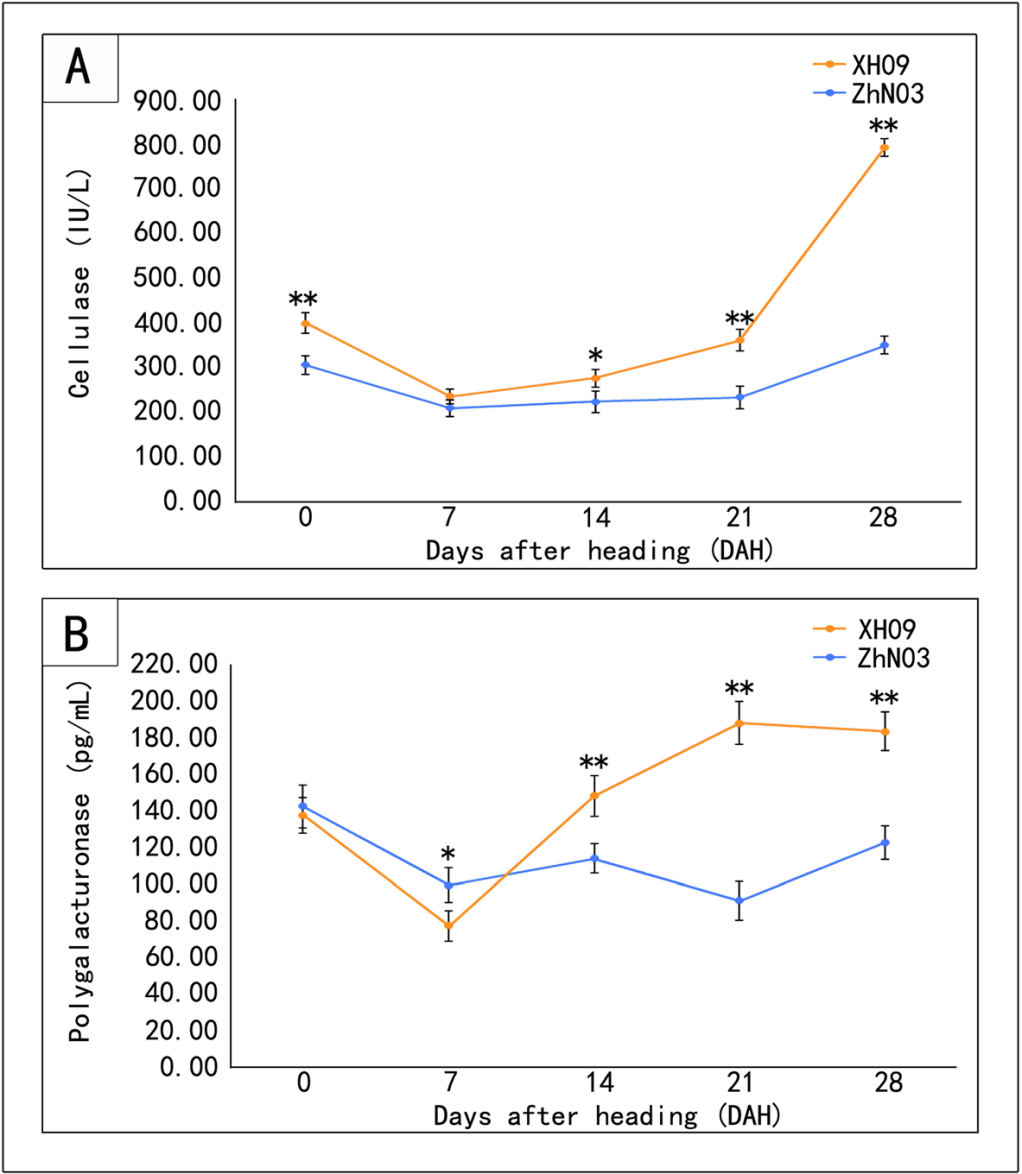
Specific activity of two cell wall-degrading enzymes: cellulase (**a**) and polygalacturonase (**b**) in abscission zone. Bars indicate the mean values ± standard deviation. Double asterisks (**) represent significant difference of enzyme activity between XH09 and ZhN03 at *p* < 0.01 level

To investigate how cell wall-degrading enzymes contribute to seed shattering, the changes of specific activity of cellulase (Fig. 3a) and polygalactouranase (Fig. 3b) were assayed in the abscission zone of XH09 and ZhN03. The two hydrolases exhibited a similar trend of activity at different stages in the growth and development of the seed. The mean specific activity of cellulase was higher in high seed shattering genotype XH09 (415.77 IU/L) than in low seed shattering genotype ZhN03 (266.8 IU/L). The activity of cellulase increased rapidly at 21 DAH in XH09, and reached 796.38 IU/L at 28 DAH while the activity of ZhN03 was 352.98 IU/L at 28 DAH. The mean specific activity of polygalactouranase was higher in XH09 (149.35 pg/ml) than in ZhN03 (115.73 pg/ml), especially at physiological maturity. At 28 DAH, polygalactouranase activity of XH09 was 186.50 pg/ml while the activity of ZhN03 was 124.77 pg/ml. Physiochemical analysis revealed significantly different cell wall-degrading enzymes activity in the abscission zone between XH09 and ZhN03 at 21DAH and 28DAH.

### Transcriptome sequencing revealed differentially expressed transcripts in abscission zone

To dissect the molecular mechanism and explore the putative genes related to seed shattering in *E. sibiricus*. cDNA libraries were constructed from abscission zone tissue RNA samples, and sequenced using an Illumina HiSeq™ 2500 platform. These Illumina data are available in the Sequence Reads Archive (SRA) with accession number SRX2617497. After cleaning and checking the read quality, high quality reads were assembled using Trinity software. The number of sequences ranged from 12.2 - 17.0 million reads per sample (Table 1). A total of 185,523 unigenes were identified, of which 86,634 unigenes were annotated in at least one database (Table 2). The expression abundance of each sample was measured. More than 30,000 differentially expressed transcripts (DETs) were detected among *E. sibiricus* libraries at three developmental stages: 7 days, 21 days and 28 days after heading, of which 1171 (476 up-regulated, 695 down-regulated), 4421 (1151 up-regulated, 2910 down-regulated), 1878 (431 up-regulated, 1447 down-regulated) were predicted from “XH09-7 vs ZhN03-7”, “XH09-21 vs ZhN03-21”, “XH09-28 vs ZhN03-28”, respectively (Table 3).

**Table 1:** Summary of the sequence data analysis

Sample	Total clean reads	Total clean nucleotides (bp)	GC%	≥Q30 (%)
XH09-7-1	14,651,268	3,843,213,534	54.75	87.35
XH09-7-2	15,688,899	3,769,876,457	55.56	88.41
XH09-7-3	15,102,813	3,809,312,291	55.14	88.23
XH09-21-1	15,655,278	3,944,283,732	55.76	88.45
XH09-21-2	15,478,709	3,899,896,658	55.46	88.61
XH09-21-3	15,122,910	3,810,402,251	55.17	88.35
XH09-28-1	14,728,212	3,710,976,309	57.68	88.03
XH09-28-2	14,400,350	3,628,304,803	57.24	88.17
XH09-28-3	14,879,668	3,749,135,994	55.56	88.26
ZhN03-7-1	14,439,791	3,822,708,203	54.61	88.57
ZhN03-7-2	13,549,381	3,410,137,265	53.86	88.48
ZhN03-7-3	13,403,148	3,447,463,715	54.14	88.54
ZhN03-21-1	15,529,892	3,912,968,243	53.71	88.59
ZhN03-21-2	13,494,783	3,400,148,238	54.36	88.58
ZhN03-21-3	13,353,208	3,364,473,812	54.17	88.64
ZhN03-28-1	12,247,393	3,085,813,765	57.48	88.67
ZhN03-28-2	13,062,771	3,291,238,868	56.90	88.73
ZhN03-28-3	17,028,238	4,290,493,634	57.88	88.45

**Table 2:** BLAST analysis of the non-redundant unigenes against public databases

Annotated database	Number of Unigene	300 ≤ length < 1000	length ≥ 1000
Nr annotation	65,838	35,264	30,574
GO annotation	44,054	20,100	23,954
Pfam annotation	42,613	15,787	26,826
KOG annotation	35,924	13,211	22,713
SwissProt annotation	44,012	21,214	22,798
KEGG annotation	23,362	10,468	12,894
COG annotation	23,512	9127	14,385
All annotated	86,634	45,380	41,254

**Table 3:** Statistical table of differently expressed transcripts (DETs), with annotation

Type	XH09-7 vs ZhN03-7	XH09-21 vs ZhN03-21	XH09-28 vs ZhN03-28
num	1171	4421	1878
up	476	1151	431
down	695	2910	1447
COG	52	766	454
GO	181	1837	571
KEGG	74	810	544
KOG	135	1249	763
Pfam	222	1958	951
SwissPort	167	1692	657
nr	435	2932	1109
all annotated	544	2974	1231

These DETs were searched against the GO database to categorize standardize gene function. A total of 2589 DETs were assigned to three main GO categories (cellular component, biological process and molecular function) and 53 subcategories (Additional file 2: Figure S1). In the cellular component category, “cell part”, “organelle”, and “membrane” were dominant groups. In the biological process category, “metabolic process”, “cellular process” and “single-organism process” were dominant groups. In the molecular function category, “catalytic activity”, “binding” and “transporter activity” were the dominant categories. To reveal the significantly enriched GO terms in the DETs, a GO enrichment analysis of the functional significance was performed via the agriGO website. 11, 70, 51 significantly enriched GO terms were found in “XH09-7 vs ZhN03-7”, “XH09-21 vs ZhN03-21”, “XH09-28 vs ZhN03-28”, respectively (Additional file 3: Table S2).

To characterize the complex biological behaviors of the transcriptome, all the DETs from three differentially expressed transcript sets were also subjected to a KEGG pathway enrichment analysis. In total, 1318 DETs could be annotated and assigned to KEGG pathway, of which 107, 512, 699 DETs were found in “XH09-7 vs ZhN03-7”, “XH09-21 vs ZhN03-21”, “XH09-28 vs ZhN03-28”, respectively (Additional file 4: Figure S2). The most representive pathway found included “ribosome (Ko03010)”, “carbon metabolism (ko01200)”, “apoptosis (Ko4210), “protein processing in endoplasmic reticulum (Ko04141)”, and so on. In this study we mainly focused on “peroxisome (Ko04146)”, “phenylpropaniod biosynthesis (Ko00940)”, “plant hormone signal transduction (Ko4075)”. Overall, in the pathway of “phenylpropaniod biosynthesis” (Additional file 5: Figure S3), 59 unigenes were annotated and encoded 12 putative enzymes involved in lignin biosynthesis. In the pathway of “plant hormone signal transduction” (Additional file 6: Figure S4), 54 unigenes were differentially expressed, of which 7 were involved in ethylene biosynthesis and regulation, 10 for abscisic acid, and 17 for auxin (Table 4).

**Table 4:** Candidate genes enriched in phenylpropanoid biosynthesis and plant hormone signal transduction pathway

KEGG pathway	Gene	Definition	KO id	EC no.	No.All^a^	No.Up^b^	No.Down^c^
Plant hormone signal transduction							
Abscisic acid	PP2C	protein phosphatase 2C	K14497	3.1.3.16	5	4	1
	SRK2	serine/threonine-protein kinase	K14498	2.7.11.1	2	2	0
	ABF	ABA responsive element binding factor	K14432		3	1	2
Ethylene	ETR	ethylene receptor	K14509	2.7.13.-	2	2	0
	EIN2	ethylene-insensitive protein 2	K14513		3	2	1
	EIN3	ethylene-insensitive protein 3	K14524		2	2	0
Auxin	AUX1	auxin influx carrier	K13946		1	0	1
	IAA	auxin-responsive protein IAA	K14484		6	1	5
	ARF	auxin response factor	K14486		2	1	1
	GH3	auxin responsive GH3 gene family	K14487		2	0	2
	SAUR	SAUR family protein	K14488		6	1	5
Cytokinine	CRE1	arabidopsis histidine kinase 2/3/4	K14489	EC:2.7.13.3	1	0	1
	AHP	histidine-containing phosphotransfer peotein	K14490		1	1	0
	B-ARR	two-component response regulator ARR-B family	K14491		3	0	3
	ARR-A	two-component response regulator ARR-B family	K14492		3	0	3
Gibberellin	TF	phytochrome-interacting factor 4	K16189		1	0	1
Brassinosteroid	BRI1	protein brassinosteroid insensitive 1	K13415	EC:2.7.10.1	1	0	1
	BSK	BR-signaling kinase	K14500	EC:2.7.11.1	1	1	0
Jasmonic acid	COI1	coronatine-insensitive protein 1	K13463		1	0	1
	JAZ	jasmonate ZIM domain-containing protein	K13464		2	0	2
Salicylic acid	NPR1	regulatory protein NPR1	K14508		3	1	2
	TGA	transcription factor TGA	K14431		3	0	3
Phenylpropanoid biosynthesis	PAL	phenylalanine ammonia-lyase	K10775	4.3.1.24	5	0	5
	4CL	4-coumarate--CoA ligase	K01904	6.2.1.12	4	0	4
	P/TAL	phenylalanine/tyrosine ammonia-lyase	K13064	4.3.1.25	1	0	1
	F5H	ferulate-5-hydroxylase	K09755	1.14.-.-	1	0	1
	CCoa-OMT	caffeoyl-CoA O-methyltransferase	K00588	2.1.1.104	2	0	2
	CALDH	coniferyl-aldehyde dehydrogenase	K12355	1.2.1.68	1	0	1
	BGLU	beta-glucosidase	K01188	3.2.1.21	8	3	5
	CCR	cinnamoyl-CoA reductase	K09753	1.2.1.44	3	2	1
	CAD	cinnamyl-alcohol dehydrogenase	K00083	1.1.1.195	5	2	3
	POX	peroxidase	K00430	1.11.1.7	21	6	15
	SOH	shikimate O-hydroxycinnamoyltransferase	K13065	2.3.1.133	6	2	4
	C3’H	Coumaroylquinate (coumaroylshikimate) 3′-monooxygenase	K09754	1.14.13.36	2	1	1

^a^the total number of uni-transcripts analysed

^b^the number of uni-transcripts with expression significantly up-regulated in high seed shattering genotype compared with low seed shattering genotype

^c^the number of uni-transcripts with expression significantly down-regulated in high seed shattering genotype compared with low seed shattering genotype

### Comparative transcriptome analysis revealed candidate transcripts involved in seed shattering

**Fig. 4:**
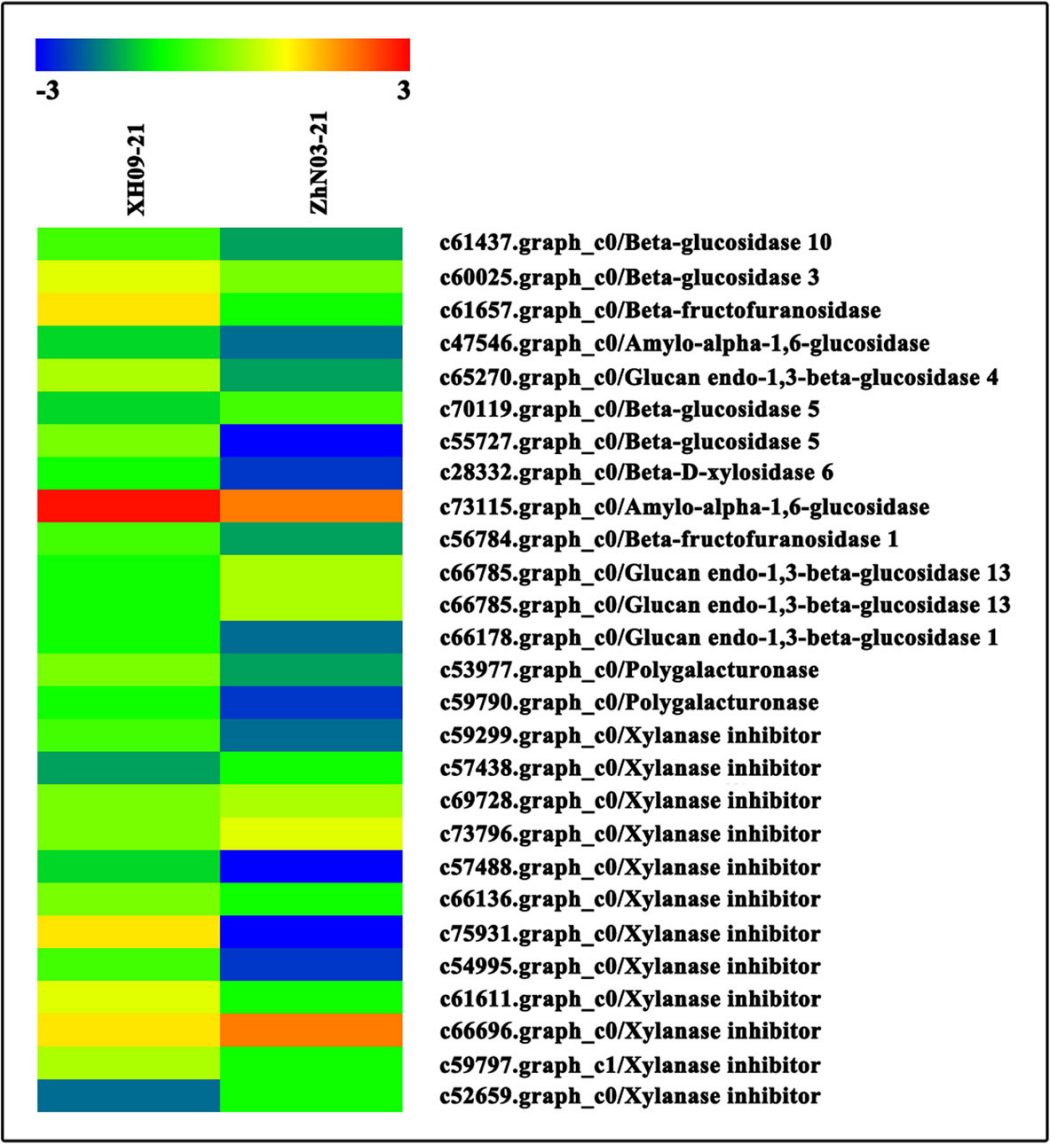
Heat map diagram of the expression levels of 27 differentially expressed transcripts (DETs) involved hydrolase activity. The DETs were found between high seed shattering genotype XH09 and low seed shattering genotype ZhN03 at 21 days after heading

Seed shattering measurement and physiochemical analysis revealed significantly different BTS values and cell wall-degrading enzymes activity in the abscission zone between XH09 and ZhN03 at 21 DAH and 28 DAH. To identify candidate genes for seed shattering, we compared the DETs in both genotypes at the three time points: 7 DAH, 21 DAH and 28 DAH. A total of 7470 DETs were detected at three developmental stages, of which 1171 DETs were predicted from “Xh09-7 vs ZhN03-7” and more DETs were predicted from “Xh09-21 vs ZhN03-21” and “Xh09-28 vs ZhN03-28”. Based on the annotation, we further selected 18, 138 and 97 putative genes in response to seed shattering from “XH09-7 vs ZhN03-7”, “XH09-21 vs ZhN03-21” and “XH09-28 vs ZhN03-28”, respectively (Additional file 7: Table S3). From the putative function of these DETs, we found 5 major function group: cell wall hydrolysis or modification, hydrolase activity, phytohormone signaling and response, transcription factor, and protein kinase activity. Eight candidate DETs involved in peroxidase activity (c60174.graph_c0), hydrolase activity (c72047.graph_c1, c30667.graph_c0, c54680.graph_c1,), ethylene-responsive transcription factor (c23015.graph_c0,), and wall-associated receptor kinase (c34865.graph_c0, c42329.graph_c0, c68413.graph_c0) were found at all the three developmental stages. A total of 58 DETs involved in hydrolase activity were predicted from “XH09-21 vs ZhN03-21”, of which 13, 2 and 12 genes involved in glucosidase activity, polygalacturonase activity and xylanase inhibitor were differentially expressed in the abscission zone of XH09 and ZhN03, respectively (Fig. 4). In particular, two genes involved in polygalacturonase activity were down-regulated in low seed shattering genotype ZhN03 compared to high seed shattering genotype XH09. In “XH09-28 vs ZhN03-28”, 18 of 97 genes were up-regulated in the abscission zone of the low seed shattering genotype ZhN03. In particular, a xylanase inhibitor protein (*XIP*) gene was expressed in the abscission zone, and an ethylene responsive transcription factor and an ethylene receptor gene (*EIN4*) involved in phytohormone signaling was up-regulated in ZhN03 at 28 DAH compared to high seed shattering genotype XH09.

### RNA-seq expression validation by quantitative reverse transcription PCR (qRT-PCR)

**Fig. 5:**
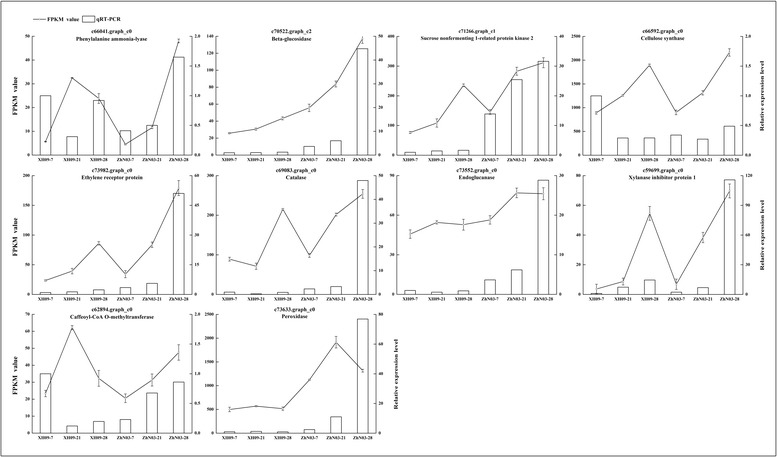
qRT-PCR validations of RNA-seq data. Expression profiles of the selected genes as determined by RNA-seq and qRT-PCR. Data were collected from high seed shattering genotype XH09 and low seed shattering genotype ZhN03 at 7, 21 and 28 days after heading. The left-hand y-axis indicates FPKM value. The right-hand y-axis indicates relative expression level. Bars indicate the mean values ± standard deviation

To quantitatively determine the reliability of our transcriptome data, ten transcripts involved in activation of abscission were selected for qRT-PCR validation. These candidates included phenylalanine ammonia-lyase (*PAL*), beta-glucosidase (*GLU*), caffeoyl-CoA O-methyltransferase (*CCoAOMT*), peroxidase (*POX*), serine/threonine-protein kinase SRK2 (*SnRK2*), ethylene receptor (*ETR*), catalase (*CAT*), Endoglucanase (*EGL*), xylanase inhibitor protein 1 (*XIP1*) and cellulose synthase (CesA). The results showed that all ten transcripts were expressed in the AZ at three development stages, 7 days, 21 day and 28 days after heading. We used XH09-7 as a benchmark for relative expression analysis. *CCoAOMT* and *CesA* were down-regulated in XH09 and ZhN03 at 21DAH and 28 DAH. The expression of other 8 transcripts was up-regulated in ZhN03-28 (Fig. 5). The relative expression of *XIP1* for ZhN03-28 was almost 120 times higher than that of XH09-07. Six genes (*GLU*, *POX*, *EGL*, *CAT*, *ETR* and *SnRK2*) were differentially expressed in ZhN03-21 and ZhN-28 in comparison of the high-seed shattering genotype XH09. A linear regression analysis of the fold-change in expression measured via RNA-seq vs qRT-PCR displayed a positive correlation (*r* = 0.76, *P* < 0.05).

## Discussion

### Histological and physiochemical difference of abscission zone

Shedding of leaves, fruit and seeds is a complex and highly coordinated process involving multiple changes in cell structure, metabolism and gene expression [32]. To elucidate the mechanism responsible for abscission in *E. sibiricus* in the present study, we conducted a combination of morphological, histological, physiochemical and transcriptome analysis in two genotypes (XH09 and ZhN03) with contrasting seed shattering phenotypes. The results showed that the high seed shattering genotype XH09 had a lower BTS value (36.73 gf) at seed physiological maturity when compared to low seed shattering genotype ZhN03 (96.3 gf). Histological analysis of abscission zone showed a smooth fracture surface of rachilla in XH09, suggesting the higher level of degradation. This may resulted from the increased cellulase and polygalacturonase activity found in abscission zone of XH09. In several systems, abscission is related to cleavage and degradation of cell wall components by cell wall hydrolytic enzymes including cellulase and polygalacturonase; and the activity of cellulase is associated with many processes of plant growth and development, such as fruit ripening and organ abscission [33]. A correlation between increasing polygalacturonase activity and cell separation was reported in plant organs [34], such that abscission-specific polygalacturonase might play an important role in breaking down the pectin rich middle lamella during the abscission process that leads to separation [32]. Our results indicated the involvement and role of cellulase and polygalacturonase in seed shattering.

### Cell wall hydrolysis related genes

The plant cell wall is mainly composed of non-starch polysaccharides, including cellulose and hemicellulose [35]. Cellulase (1,4,-*β*- glucanase) is the first enzyme reported to contribute to wall loosening during abscission [36]. Our KEGG pathway enrichment analysis of the DETs indicated 28 unigenes involved in cellulase activity. Most of these unigenes were up-regulated in the abscission zone of both genotypes at 28 days after heading. These results indicated that higher expression of these unigenes might lead to an increase in seed shattering at seed physiological maturity. Many plant cellulase genes belong to a glycosyl hydrolase family that modify cell wall structure and component during tissue development [37, 38]. In rice, the gene *OsCel9D* (synonym *OsGLU1*), encoding an endo-1,4,-*β*- glucanase gene with cellulose function, is related to the cell wall components in rice; and *OsCel9D* mutations reduce cell elongation and cellulose content, and increase the pectin content, therefore hampering the abscission process in seed shattering [38]. The relative expression of an endo-1,4-*β*-glucanase gene in rice was found to be associated with seed shattering [39]*.* During the abscission of leaves, flowers and seeds, increased expression of endo-1,4-*β*-glucanase gene could facilitate natural separation of plant organs [40–42].

Xylan is the major component of hemicelluloses. Xylanase can catalyze the hydrolysis of the *β*-1,4-xylosidic bonds in xylan, the activity of xylanase can be inhibited by xylanase inhibitors (*XIs*) [35]. Xylanases have been reported to play an important role in plant defense against pathogens [43] and herbivores [35]. However, whether xylanases are also involved in seed shattering remains largely unknown. In rice, at least three XIP type xylanase inhibitor genes (*rice XIP*, *RIXI* and *OsXIP*) have been reported, and these genes are differentially induced by stress [44–46]. In the present study, 12 *XIP* genes were differently expressed in the AZ of both genotypes at 21 DAH (Fig. 4), and the low shattering genotype ZhN03 showed much higher expression of these genes when compared with high shattering genotype XH09. This indicates that this gene might have an effect on seed shattering in the evaluated genotypes, and the expression of this gene is associated with a reduction of seed shattering.

### Plant hormone-related genes

Plant hormones, also known as phytohormones, are signal molecules produced within the plant that have an important role in regulating a wide range of plant growth and development processes, including abscission. Our KEGG pathway enrichment analysis of the DETs indicated 54 unigenes involved in plant hormone signal transduction, of which 17 were related to Auxin, 8 to Cytokinine, 1 to Gibberellin, 10 to Abscisic acid, 7 to Ethylene, 2 to Brassinosteroid, 3 to Jasmonic acid, and 6 to Salicylic acid response pathways. Abscisic acid, ethylene, and auxin are important plant growth regulators in regulating abscission [32, 47]. Abscisic acid plays a direct role in abscission of organs such as seeds [48]. Abscisic acid signal transduction is regulated by several groups of ABA-responsive genes such as an ABA receptor *PYR/PYL*, a type 2C protein phosphatase (*PP2C*), a serine/threonine protein kinase (*SnRK2*) and an ABRE-binding factor (*ABF*) [49–51]. Previous studies have shown that *PP2Cs* are negative regulators of ABA signaling [49]. On the other hand, *SnRK2* positively regulate ABA responses [50], but its activity can be inhibited by *PP2C*. In the presence of ABA, the interaction between the *PP2Cs* and *SnRK2s* can be disturbed by the *PYR/PYL* receptor, thus preventing the *PP2C*-mediated dephosphorylation of *SnRK2*, causing the activation of *SnRK2* kinases [51]. In the present study, we found four of the five *PP2C* genes up-regulated, two *SnRK2* genes up-regulated, and one of two identified *ABF* genes up-regulated in the abscission zone of the low seed shattering genotype. Our results suggest the interaction of these ABA-responsive genes may have contributed to seed shattering.

Ethylene is an important plant hormone also known to regulate flower and seed abscission, and elevation in ethylene production is commonly associated with tissue senescence and cell stress [52]. In the present study, we found that 6 ethylene-responsive genes (2 *ETR* genes, 2 *EIN2* gene and 2*EIN3* genes) were up-regulated in abscission zone of low seed shattering genotype. Several homologs of these genes have been involved in senescence in *Arabidopsis* and tomato, including *ETR1* [53] and its homologous genes *eTAEl* [54], *LeETR1* and *LeETR2* [55], *ERS* [56, 57], and *EIN3/EIL* [58]. The ethylene insensitive mutant of *Arabidopsis etr1* exhibited a delay in the shedding of floral parts, suggesting the roles in regulating the timing of abscission.

As with ethylene responses, many genes required for normal auxin signaling have been identified, including *AUX/IAA*, the small auxin up RNA (*SAUR*), and gretchehagen-3 (*GH3*) [59]. In this study, three IAA responsive genes (1 *SAUR*, 1 *ARF*, and 1 *AUX/IAA*) were up-regulated in the abscission zone of our low seed shattering genotype. In rice, overexpression of a *SAUR* gene caused reductions in root and shoot growth and development, indicating it functions as a negative regulator of auxin synthesis and transport [60]. *GH3*, as a negative feedback regulator of IAA concentration, can help maintain auxin homeostasis [61]. Additionally, ethylene is a potent inhibitor of auxin while the auxin level of the abscission zone significantly affects the sensitivity to ethylene [32]. A balance and interaction between ethylene and auxin (IAA) may be the key factor that regulates and determine the timing of the abscission process.

### Lignin biosynthesis related genes in the AZ are putative seed shattering genes

Lignin is a complex phenylpropanoid polymer, fills the spaces between cell wall polysaccharides, and confers mechanical strength to the cell wall [62]. It is identified as a major factor in the recalcitrance of cell walls to digestion, particularly during enzymatic hydrolysis [63]. A previous study in rice showed that seed shattering can be induced by inhibiting lignin biosynthesis, where overexpression of the BEL1-type homeobox gene *SH5* in the non-shattering “IIpum” variety led to an increase in seed shattering because lignin levels were decreased in the abscission zone and surrounding pedicel tissues [64]. In present study, staining of pedicels at 21 days and 28 days after heading showed that lignin deposition was much lower in XH09 than in ZhN03. Meanwhile, XH09 had lower BTS value when compared with ZhN03. These results implied high seed shattering degree of XH09 may be due to a reduction of lignin content. At least ten enzymes are required for monolignol biosynthesis: phenylalanine ammonia-lyase (*PAL*), cinnamic acid 4-hydroxylase (*C4H*), cinnamyl-alcohol dehydrogenase (*CAD*), cinnamoyl-CoA reductase (*CCR*), caffeic acid/5-hydroxyferulic acid *O*-methyltransferase (*CoMT*), caffeoyl-CoA O-methyltransferase (*CCoAOMT*), coniferaldehyde dehydrogenase (*CALDH*), *p*-coumarate: CoA ligase (*4CL*), ferula 5-hydroxylase (*F5H*) and shikimate O-hydroxycinnamoy transferase (*HCT*) [65]. Generally, suppression of genes early in the monolignol biosynthetic pathway, such as *PAL*, *C4H*, *HCT* and *C3’H*, significantly reduce lignin content [63, 66]. A similar result was found in this study, where *PAL* was down-regulated and lignin content was lower in low seed shattering genotype XH09, corresponding to increased seed shattering. Changes in the expression level of other monolignol biosynthesis genes affect the amount of lignin and lignin composition [62, 67, 68]. In the present study, two *CAD* genes were down-regulated in XH09. Expression of genes in the monolignol biosynthetic pathway can also be regulated by many transcription factors with a MYB DNA binding domain [69, 70]. We found two transcription factors with MYB-like DNA binding domains that were differently expressed in XH09 and ZhN03 at seed physical maturity; one was up-regulated in ZhN03 while the other was down-regulated. These results indicated that the different expression patterns of these identified DETs may resulted in the difference of lignin content in abscission zone and surrounding pedicel tissues, that may affect the seed shattering degree of XH09 and ZhN03.

## Conclusions

Seed shattering of *E. sibiricus* is caused by a degradation of the abscission layer formed at the basal part of grains. High seed shattering genotype XH09 had higher activity of cellulase and polygalacturonase in the abscission zone. In present study more than 30,000 DETs were detected among the *E. sibiricus* libraries, of which 7.470 DETs were predicted from “XH09-7 vs ZhN03-7”, “XH09-21 vs ZhN03-21” and “XH09-28 vs ZhN03-28”*.* Many genes that involved in cell wall-degrading enzymes, lignin biosynthesis, and plant hormones (e.g. ethylene, auxin and abscission acid) were differentially transcribed. The expression of some genes (e.g., *PAL*, *ABF*, *XIP* and *EGL*) were associated with reduction of seed shattering, but which genes played a key role in difference of seed shattering still remains unknown. These transcripts provide hypotheses for further testing and development of low-shatter *E. sibiricus* germplasm. This study provided novel insights into the mechanism of seed shattering in *E. sibiricus.*

## Additional files

Additional file 1: Table S1. Primers used for qRT-PCR analysis. (XLS 22 kb)

Additional file 2: Figure S1. GO classification results of differentially expressed transcripts (DETs) found in three DETs sets. The genes were assigned to three main categories: cellular component, molecular function and biological process. The right-hand y-axis indicates the number of annotated genes. The left-hand y-axis indicates the percentage of annotated genes. (PDF 617 kb)

Additional file 3: Table S2. Significantly-enriched GO terms found in three differentially expressed transcript sets. (XLS 49 kb)

Additional file 4: Figure S2. KEGG classification results of differentially expressed transcripts (DETs) found in three DETs sets. All DETs were assigned to five categories: cellular process, environmental information processing, genetic information processing, metabolism and organismal systems. (PDF 182 kb)

Additional file 5: Figure S3. Differentially expressed transcripts involved in the monolignol biosynthesis. Green means that the DETs were down regulated, red represents up regulated, and blue indicates that genes were of mixed expression patterns in the low seed shattering genotype compared to the high seed shattering genotype. (PDF 67 kb)

Additional file 6: Figure S4. Differentially expressed transcripts involved in plant hormone signal transduction pathway. Green means that the DETs were down regulated, red represents up regulated, and blue indicates that genes were of mixed expression patterns in the low seed shattering genotype compared to the high seed shattering genotype. The diagram of network was cited from KEGG website. (PDF 176 kb)

Additional file 7: Table S3. Differentially expressed transcripts related to seed shattering found in three DETs sets at three time points. (XLS 567 kb)

## Abbreviations

AZ: Abscission zone
BTS: Breaking tensile strength
CE: Cellulase
COG: Cluster of orthologous group
DAH: Days after heading
DET: Differentially expressed transcript
FDR: False discovery rate
FPKM: Fragments per kilobase per million fragments mapped
GO: Gene ontology
KEGG: Kyoto encyclopedia of genes and genomes
KOG: euKaryotic orthologous groups
NGS: Next-generation sequencing
Nr: Non-redundant protein sequence
Pfam: Protein family
PG: Polygalacturonase
qRT-PCR: Quantitative real-time PCR
QTL: Quantitative trait locus
RNA-Seq: Ribonucleic acid sequencing
SNP: Single nucleotide polymorphism
Swiss-Prot: Annotated protein sequence database
