# Supplementary material for: Transcriptome profiling of Elymus sibiricus, an important forage grass in Qinghai-Tibet plateau, reveals novel insights into candidate genes that potentially connected to seed shattering
Source: BMC Plant Biol. 2017 Apr 21;17:78. doi: 10.1186/s12870-017-1026-2 (PMC5399857; doi:10.1186/s12870-017-1026-2)
Supplement: Supplementary file 2 — GO classification results of differentially expressed transcripts (DETs) found in three DETs sets. The genes were assigned to three main categories: cellular component, molecular function and biological process. The right-hand y-axis indicates the number of annotated genes. The left-hand y-axis indicates the percentage of annotated genes. (PDF 617 kb) [file 12870_2017_1026_MOESM2_ESM.pdf]

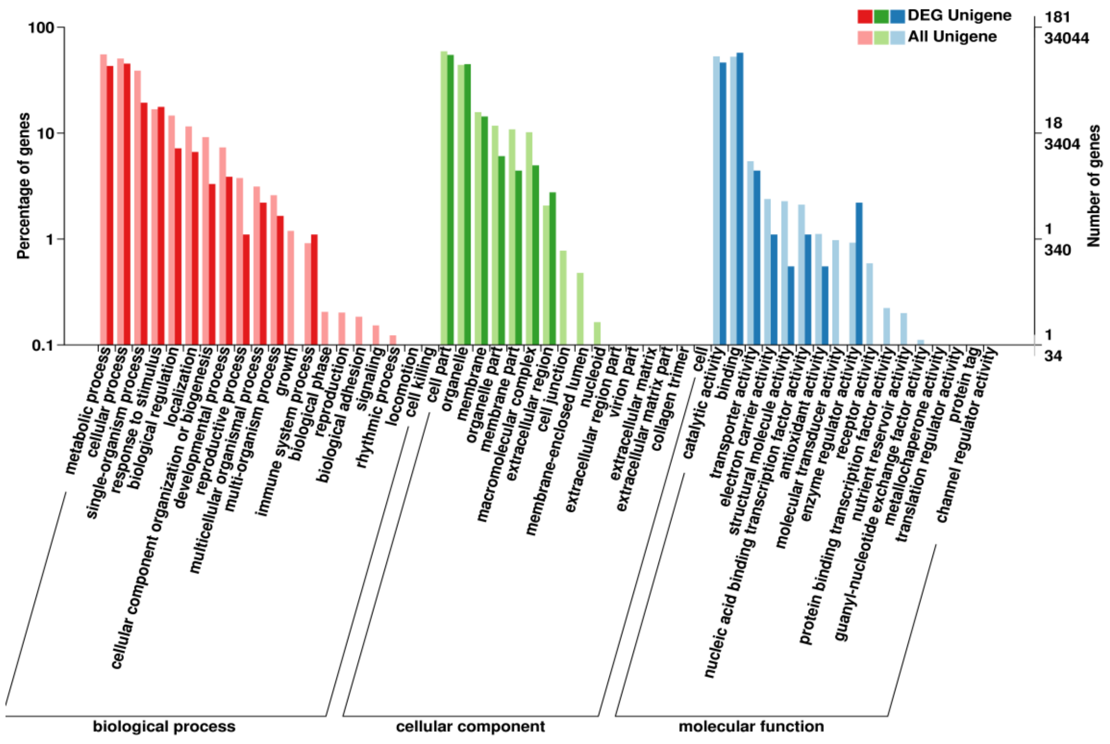

A: GO classification results of differentially expressed Transcripts (DETs) found in XH09-7 vs ZhN03-7

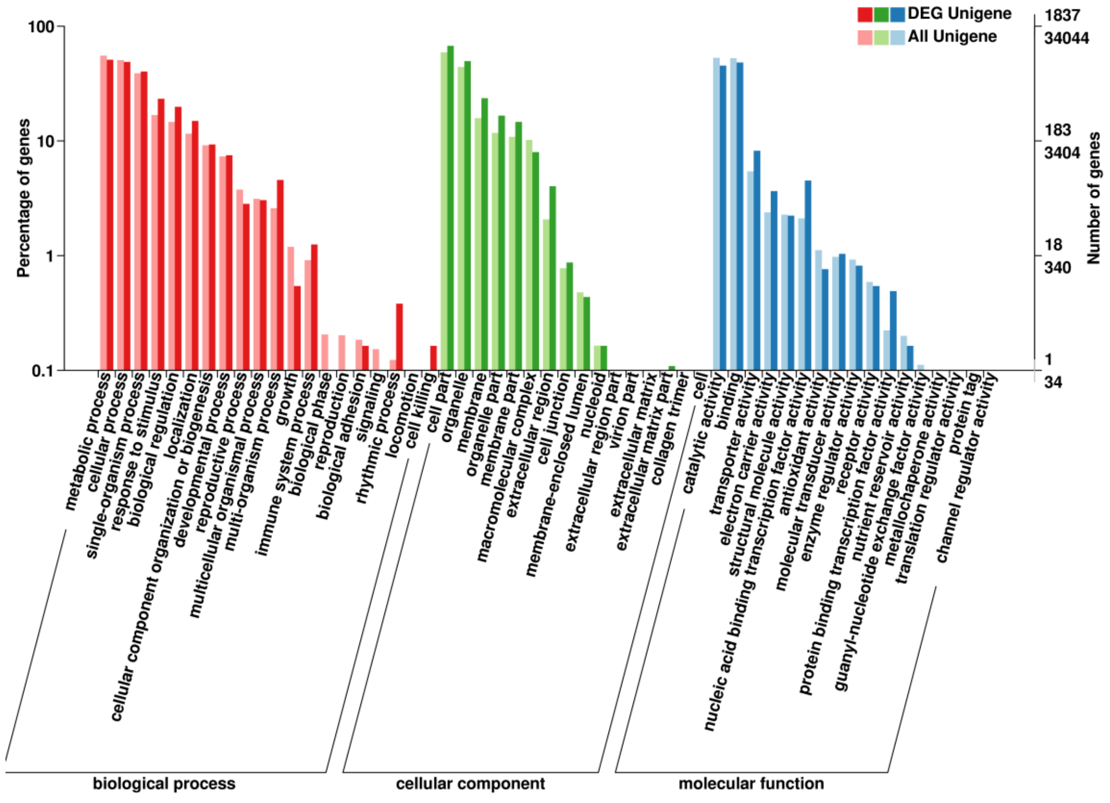

B: GO classification results of differentially expressed Transcripts (DETs) found in XH09-21 vs ZhN03-21

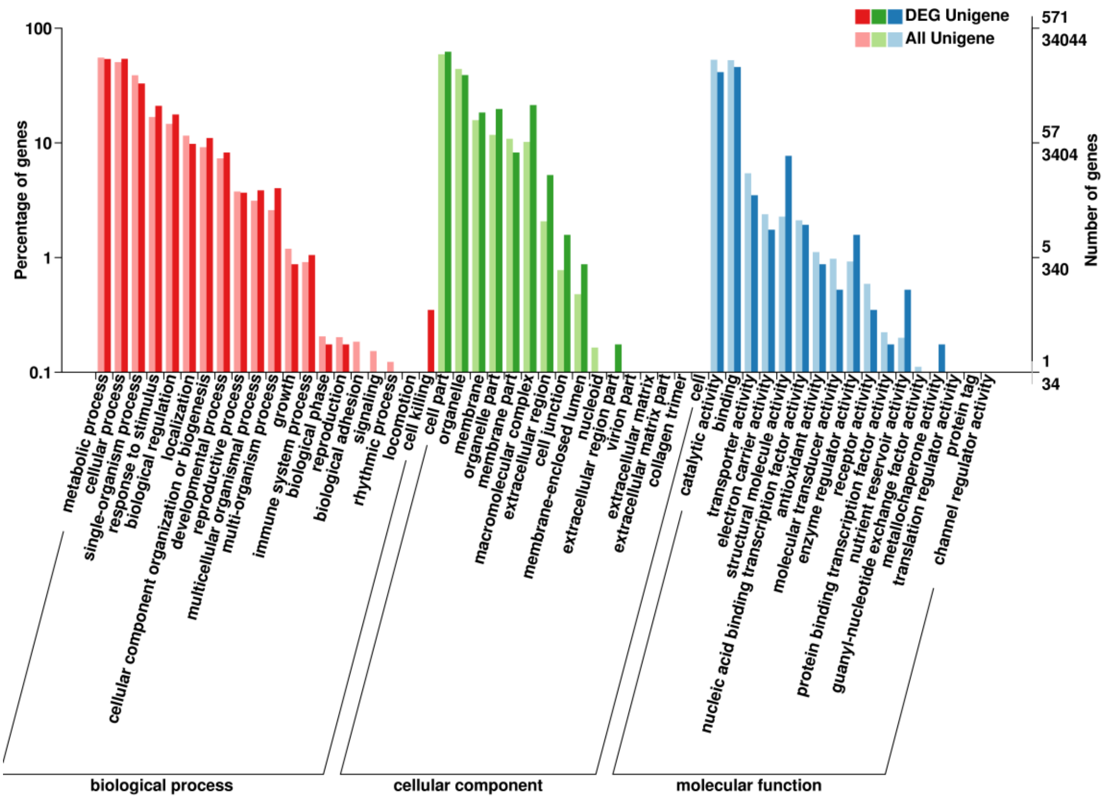

C: GO classification results of differentially expressed Transcripts (DETs) found in XH09-28 vs ZhN03-28

Figure S1. GO classification results of differentially expressed Transcripts (DETs) found in three DETs sets. The genes were assigned to three main categories: cellular component, molecular function and biological process. The right-hand y-axis indicates the number of annotated genes. The left-hand y-axis indicates the percentage of annotated genes.
