# Supplementary material for: Transcriptome profiling of Elymus sibiricus, an important forage grass in Qinghai-Tibet plateau, reveals novel insights into candidate genes that potentially connected to seed shattering
Source: BMC Plant Biol. 2017 Apr 21;17:78. doi: 10.1186/s12870-017-1026-2 (PMC5399857; doi:10.1186/s12870-017-1026-2)
Supplement: Supplementary file 4 — KEGG classification results of differentially expressed transcripts (DETs) found in three DETs sets. All DETs were assigned to five categories: cellular process, environmental information processing, genetic information processing, metabolism and organismal systems. (PDF 182 kb) [file 12870_2017_1026_MOESM4_ESM.pdf]

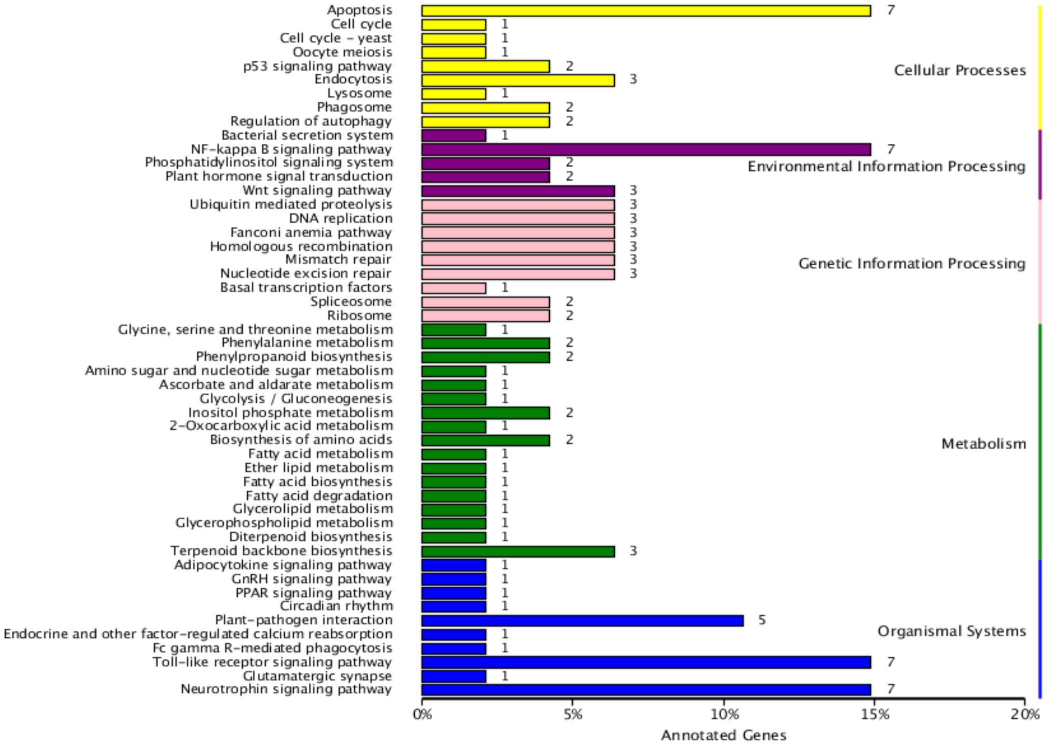

A: KEGG enrichment of DETs from XH09-7 vs ZhN03-7

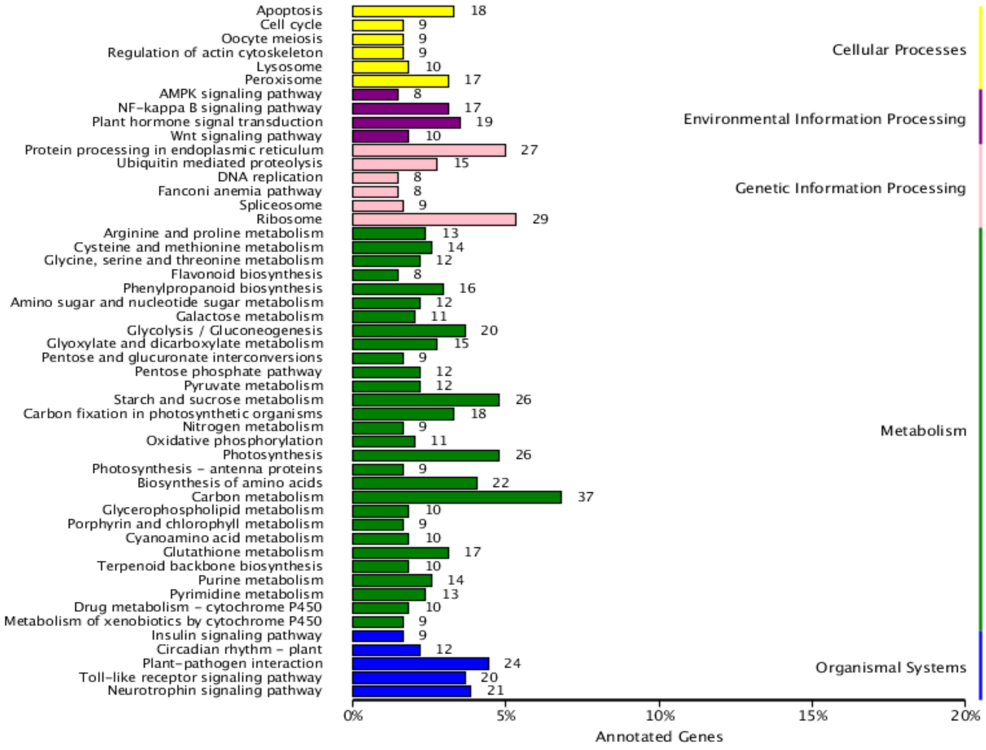

B: KEGG enrichment of DETs from XH09-21 vs ZhN03-21

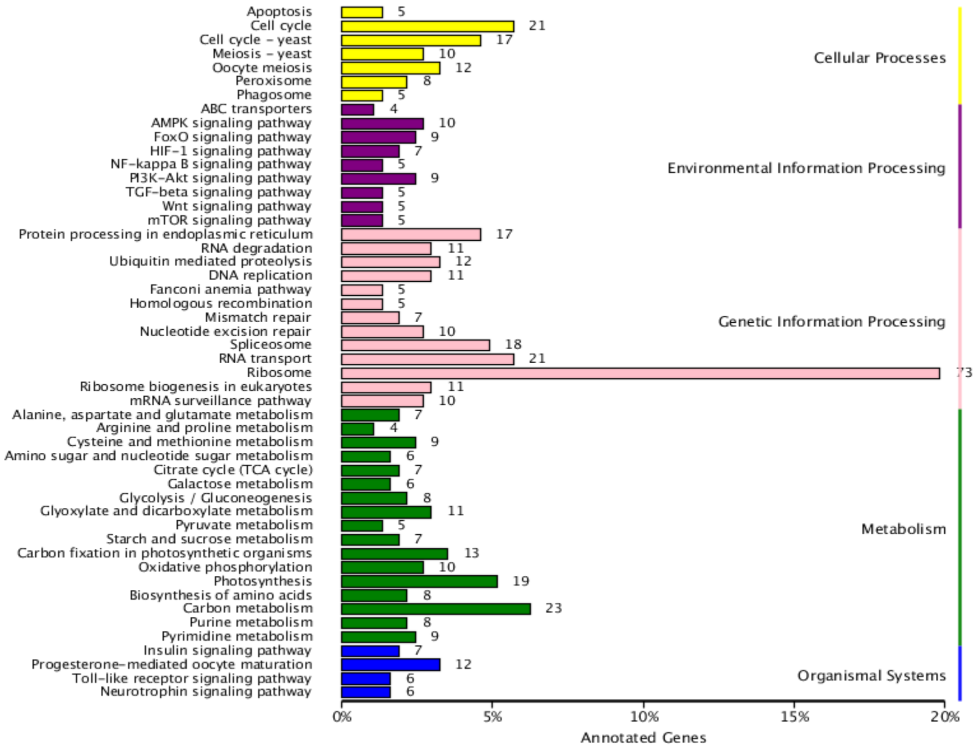

C: KEGG enrichment of DETs from XH09-28 vs ZhN03-28

Figure S2. KEGG classification results of differentially expressed transcripts (DETs) found in three DETs sets. All DETs were assigned to five categories: cellular process, environmental information processing, genetic information processing, metabolism and organismal systems.
