# Supplementary material for: Transcriptome profiling of Elymus sibiricus, an important forage grass in Qinghai-Tibet plateau, reveals novel insights into candidate genes that potentially connected to seed shattering
Source: BMC Plant Biol. 2017 Apr 21;17:78. doi: 10.1186/s12870-017-1026-2 (PMC5399857; doi:10.1186/s12870-017-1026-2)
Supplement: Supplementary file 5 — Differentially expressed transcripts involved in the monolignol biosynthesis. Green means that the DETs were down regulated, red represents up regulated, and blue indicates that genes were of mixed expression patterns in the low seed shattering genotype compared to the high seed shattering genotype. (PDF 67 kb) [file 12870_2017_1026_MOESM5_ESM.pdf]

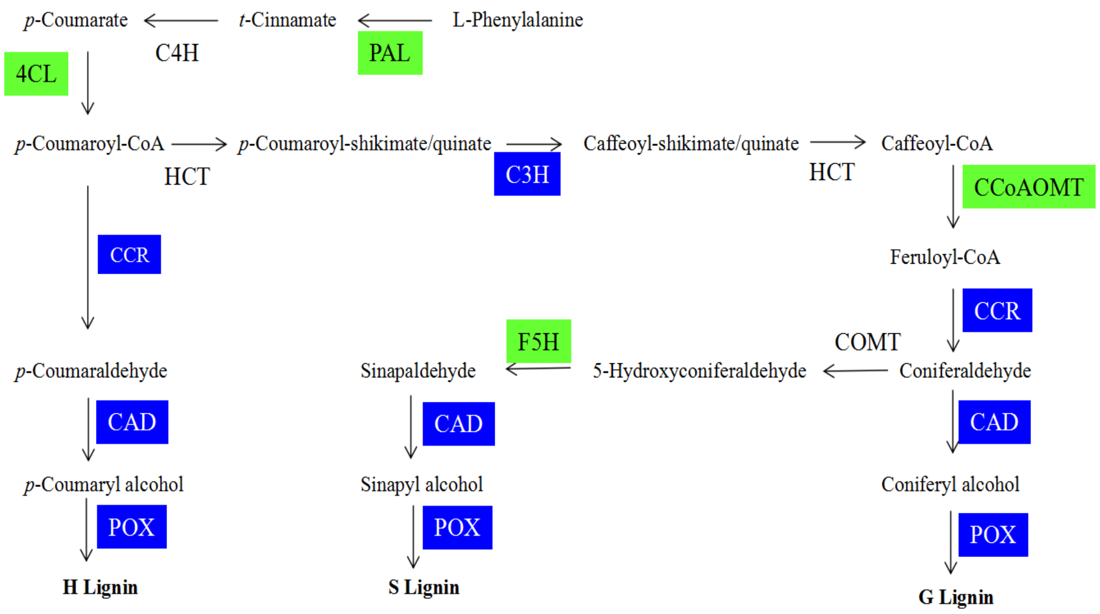

Figure. S3 Differentially expressed transcripts (DETs) involved in the monolignol biosynthesis. Green means that the DETs were down regulated, red represents up regulated, and blue indicates that genes were of mixed expression patterns in the low seed shattering genotype compared to the high seed shattering genotype.
